# Tonal language experience facilitates the use of spatial cues for segregating competing speech in bimodal cochlear implant listeners

**DOI:** 10.1121/10.0025058

**Published:** 2024-03-01

**Authors:** Biao Chen, Xinyi Zhang, Jingyuan Chen, Ying Shi, Xinyue Zou, Ping Liu, Yongxin Li, John J. Galvin, Qian-Jie Fu

**Affiliations:** 1 Department of Otolaryngology, Head and Neck Surgery, Beijing TongRen Hospital, Capital Medical University, Ministry of Education of China, Beijing, People's Republic of China; 2 House Institute Foundation, Los Angeles, California 90057, USA; 3 Department of Head and Neck Surgery, David Geffen School of Medicine, University of California, Los Angeles, Los Angeles, California 90095, USA entchenbiao@qq.com, zhangxinyitong@163.com, jyuan93@126.com, afly4448@163.com, zxy971022@126.com, liuping117266@163.com, entlyx@sina.com, jgalvin@hifla.org, qfu@mednet.ucla.edu

## Abstract

English-speaking bimodal and bilateral cochlear implant (CI) users can segregate competing speech using talker sex cues but not spatial cues. While tonal language experience allows for greater utilization of talker sex cues for listeners with normal hearing, tonal language benefits remain unclear for CI users. The present study assessed the ability of Mandarin-speaking bilateral and bimodal CI users to recognize target sentences amidst speech maskers that varied in terms of spatial cues and/or talker sex cues, relative to the target. Different from English-speaking CI users, Mandarin-speaking CI users exhibited greater utilization of spatial cues, particularly in bimodal listening.

## 1. Introduction

In listeners with normal hearing (NH), segregation of competing speech is facilitated by talker sex differences between target and masker speech (“talker sex cues”) and/or spatial separation of target and masker speech (“spatial cues”) (Kidd *et al.*, 2016). The degraded spectro-temporal resolution associated with cochlear implants (CIs) and hearing aids may limit access to talker sex and/or spatial cues for bilateral (CI in both ears) and bimodal CI users (low-frequency acoustic hearing in one ear, CI in the other ear). Previous studies have shown that English-speaking CI users may benefit from talker sex cues to segregate competing speech, but the benefit is much smaller than that of NH listeners [e.g., Cullington and Zeng (2008) and Willis *et al.* (2021)].

Significant benefits from spatial cues have been observed in bilateral [e.g., Litovsky *et al.* (2009), Gifford *et al.* (2014), and Gifford *et al.* (2018)] or bimodal CI users (Gifford *et al.*, 2014; Gifford *et al.*, 2018) in terms of head shadow effects. However, the head shadow benefit may depend on the listening devices and/or spatial configuration. For example, Gifford *et al.* (2018) found that bilateral CI users have access to head shadow benefit with both ears, whereas bimodal CI users have head shadow benefit only in the CI ear, due to the poorer performance in the non-CI ear given its limited audible bandwidth (i.e., performance asymmetry across ears). When head-shadow effects are minimized with symmetrically placed speech maskers, little or even negative masking release with spatial cues has been reported [e.g., Hu *et al.* (2018) and Willis *et al.* (2021)]. This deficit may be due to differences in spectro-temporal resolution across ears, differences in the amplitude mapping across hearing devices, frequency mismatch across ears, better-ear effects, and poor perception of inter-aural time and/or level differences.

Language experience may also affect utilization of talker sex and/or spatial cues for segregating competing speech. Zhang *et al.* (2020) found that NH tonal language speakers (Mandarin) were better able to segregate competing speech using talker sex cues than were NH non-tonal language speakers (English). Better pitch perception and better sensitivity to dynamic fundamental frequency (F0) cues due to long-term experience with tonal language [e.g., Xie and Myers (2015) and Deroche *et al.* (2019)] may have contributed to the larger masking release with talker sex cues in Mandarin-speaking listeners. Zhang *et al.* (2020) also reported that mean masking release with spatial cues was also larger in Mandarin-speaking (13.71 ± 2.70 dB) than in English-speaking NH listeners (12.23 ± 2.77 dB). However, it is unclear whether tonal language experience will similarly affect masking release with talker sex and/or spatial cues in bilateral and bimodal CI users.

The present study measured recognition of target sentences in the presence of two co-located or spatially separated speech maskers having the same or different sex as the target in Mandarin-speaking bilateral and bimodal CI. We predicted that Mandarin-speaking bilateral and bimodal CI users would be able to segregate competing speech using talker sex cues but not spatial cues, consistent with previous data from English-speaking CI listeners (Willis *et al.*, 2021). We also predicted that Mandarin-speaking CI users would experience greater masking release with talker sex and/or spatial cues than would English listeners [as with the NH listeners in Zhang *et al.* (2020)], especially for Chinese bimodal CI users where access to pitch cues may be available with acoustic hearing in the non-implanted ear.

## 2. Methods

### 2.1 Participants

Nineteen CI users participated in the study (7 male and 12 females; 11 bilateral, 8 bimodal). The mean age at testing was 28.7 years (range = 14–58 years) and the mean CI experience was 4.6 years (range = 1.0–23.1 years). For bimodal CI users, aided warble-tone thresholds were measured in sound field using participants' clinically programmed hearing aids; pure-tone average (PTA) thresholds across 250, 500, and 1000 Hz from 33 to 58 dB HL. Demographic information for the CI participants is shown in Table 1. All participants were native Chinese speakers of Mandarin. In compliance with ethical standards for human subjects, written informed consent was obtained from all participants before proceeding with any of the study procedures; in the case of the three 14-year-old BCI participants, written consent was also obtained from their parents. This study was approved by the Ethics Committee of the Beijing Tongren Hospital, Capital Medical University (TRECKY-2019-055-XZ-1).

**Table 1.** Demographic information for bilateral (top) and bimodal CI users (bottom). Age test = age at testing (years); CI exp  = years of CI experience after implantation; BI = bilateral CI; BM = bimodal CI; PTA = Pure-tone average threshold in the non-CI ear across 0.25, 0.5, and 1 kHz.

			Left Ear		Right Ear	
Participant	Sex	Age test	CI device	CI exp	CI device	CI exp
BI1^a^	M	21	Med-EL	3.0	Med-EL	3.0
BI2	M	14	Med-EL	8.5	Med-EL	2.2
BI3	F	14	Med-EL	10.9	Med-EL	2.6
BI4^a^	F	23	Med-EL	2.2	Med-EL	2.2
BI5^a^	M	18	Med-EL	4.5	Med-EL	4.5
BI6^a^	F	14	Med-EL	1.0	Med-EL	1.0
BI7	F	26	Med-EL	21.4	Med-EL	23.1
BI8	F	31	Med-EL	1.4	Med-EL	3.6
BI9	F	25	Med-EL	1.1	Med-EL	6.2
BI10	F	24	Med-EL	1.6	Med-EL	14.8
BI11^a^	F	49	Med-EL	1.1	Med-EL	1.1

			CI ear		Non-CI ear	
Participant	Sex	Age test	CI device	CI exp	HA device	PTA (dB HL)
BM1	F	30	Med-EL	2.5	Resound	43.3
BM2	F	53	Med-EL	1.3	Phonak	45.0
BM3	M	42	Med-EL	2.1	Starkey	46.7
BM4	M	18	Med-EL	2.1	Resound	35.0
BM5	M	27	Med-EL	1.8	Phonak	43.3
BM6	F	35	Med-EL	1.6	Oticon	58.3
BM7	F	58	Med-EL	4.0	Phonak	36.7
BM8	M	24	Med-EL	1.9	Resound	33.3

^a^ For bilateral CI users, the footnotes indicate simultaneous cochlear implantation; the remaining bilateral CI users were implanted sequentially.

### 2.2 Test materials and methods

The matrix-style test materials were drawn from the Closed-set Mandarin Speech corpus (Tao *et al.*, 2017; Tao *et al.*, 2018). Target and masker stimuli consisted of five-word sentences, designed according to matrix-styled test paradigms. To create the target sentences, one of 10 words was chosen at random from each of five categories (Name, Verb, Number, Color, and Object); the target speech was produced by a single male talker (mean F0 across all 50 words: 139 Hz). Similarly, masker sentences were created by choosing one of 10 words from each category that was not used for the target sentence; masker sentences each contained unique words. Masker sentences were produced by two male talkers that were different from the target male talker (mean F0s: 143 and 178 Hz), or by two female talkers (mean F0s: 208 and 248 Hz).

Speech recognition thresholds (SRTs), defined as the target-to-masker ratio (TMR) that produced 50% correct recognition of target keywords in sentences, were adaptively measured with participants wearing both devices (CI in each ear for bilateral CI users, CI in one ear and hearing aid in the other ear for bimodal CI users). Participants were tested using their clinical CI and/or hearing aid settings, which were not changed during testing. The target sentence originated directly in front of the listener (0°), and the two masker sentences were either co-located with the target (0°) or presented to the left (–90°) and right of the target (+90°) separately. The masker talker sex was either the same as or different from the male target talker. Accordingly, there were four segregation cue conditions: (1) Baseline (Base; no talker sex or spatial cues), (2) Talker sex cues (T), (3) Spatial cues (S), and (4) combined Talker sex and Spatial cues (T + S). All stimuli were presented and responses were collected using custom software [Mandarin Angel Sound software; freely available online (Emily Shannon Fu Foundaton, 2012)].

The target sentence was always presented at 65 dBA while the level of masker sentences was globally adjusted according to the correctness of the listener's response. For example, for a TMR of +10 dB, the level of the target sentence was 65 dBA and the level of each masker sentence was 55 dBA. Participants were instructed to listen to the target sentence (produced by the male target talker and beginning with the name “Xiaowang”) and then click on one of the ten response choices from each of the Number and Color categories; no selections could be made from the remaining categories which were greyed out. If the participant identified both key words correctly, the TMR was reduced; if the participant did not identify both key words, the TMR was increased. The initial step size was 4 dB and the final step size was 2 dB. The SRT was calculated by averaging the last 6 reversals in TMR. If there were fewer than 6 reversals within 20 trials, the test run was discarded and another run was measured. Two test runs were completed for each listening condition and the SRT was averaged across runs. The listening conditions and test runs were randomized within and across participants.

## 3. Results

**Fig. 1.**
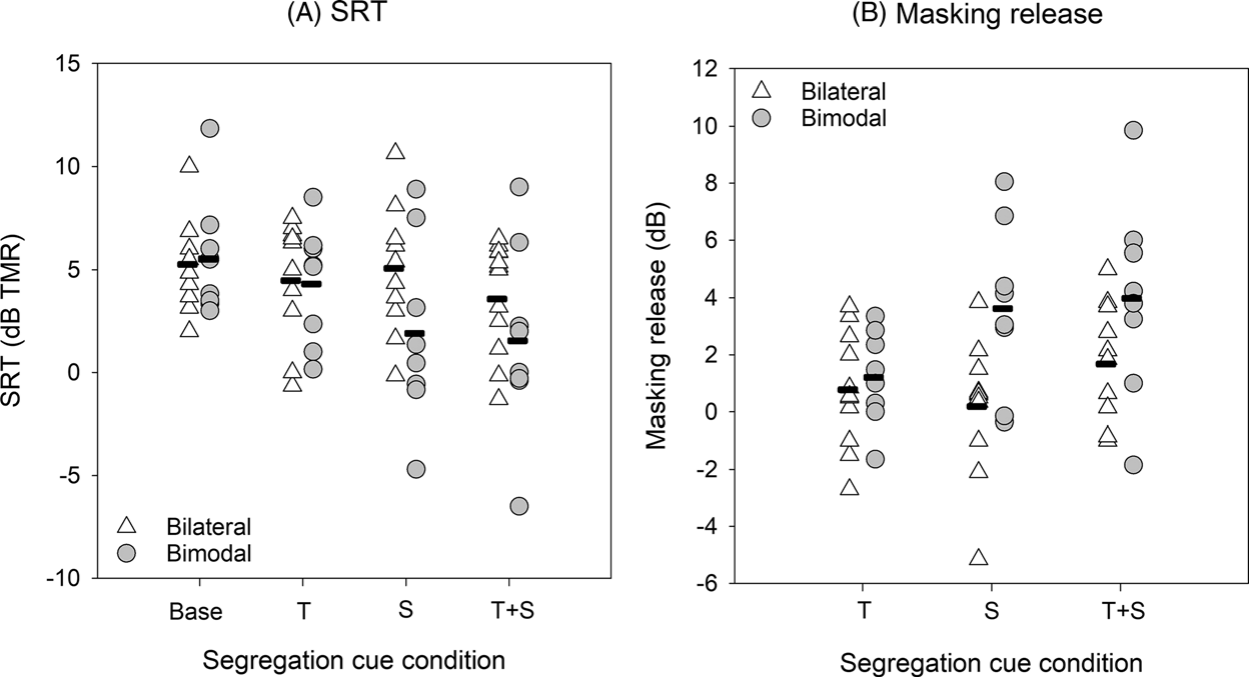
(A) SRTs for the Baseline (Base), Talker sex (T), Spatial (S), and Talker sex + Spatial (T + S) cue conditions for bilateral and bimodal CI users. (B) Masking release for the T, S, and T + S cue conditions for bilateral and bimodal CI users. Masking release was calculated relative to the Base condition. In both panels, the horizontal dashes show mean performance.

Figure 1(A) shows SRTs for bilateral and bimodal CI users and for the segregation cue conditions; mean SRTs are shown in Table 2. SRTs were generally higher for the bilateral than for the bimodal group. For both bilateral and bimodal CI users, mean SRTs were highest (poorest) for the Base condition and lowest (best) for the T + S condition. Linear mixed model (LMM) analysis was performed on the SRT data, with group (bilateral, bimodal) and segregation cue (Base, T, S, T + S) as fixed factors and participant as the random factor; complete results are shown in Table 2. Results showed a significant effect for segregation cue [F(3,51) = 9.25, p < 0.001] but not for group [F(1,17) = 0.94, p = 0.346]; there was a significant interaction [F(3,51) = 4.07, p = 0.011]. *Post hoc* Bonferroni-adjusted pairwise comparisons showed that SRTs were significantly higher for bilateral than for bimodal CI users only for the S condition (p < 0.05). SRTs were significantly higher for the Base and T conditions than for the S and T + S conditions only for the bimodal group (p < 0.05). When we excluded outlier baseline for performance for BI1 and BM5, LMM results were nearly unchanged.

**Table 2.** Top: SRTs (mean ± standard deviation) for the segregation cue conditions and CI groups. Bottom: Results from linear mixed model analysis of SRT data. Significant differences for pairwise comparisons are shown at right (adjustment for multiple comparisons: Bonferroni). BI = bilateral CI users; BM: bimodal CI users; Base = Baseline (no talker sex or spatial cues); T = Talker sex; S = Spatial; T + S: Talker sex + Spatial.

Mean SRTs (dB TMR)				
Segregation cue condition				
Group	Base	T	S	T + S
BI	5.26 ± 2.12	4.48 ± 2.77	5.07 ± 3.02	3.58 ± 2.70
BM	5.52 ± 2.95	4.31 ± 2.86	1.90 ± 4.50	1.54 ± 4.68

Linear mixed model				
	dF, res	*F*	*p*	Post-hoc (p < 0.05)
Group	1, 17	0.94	0.346	
Cue	3, 51	9.25	0.001^a^	Base, T > S, T + S
Group × cue	3, 51	4.07	0.011^a^	S: BI > BM
				BM: Base, T > S, T + S

^a^ Significant effects.

Masking release was calculated in terms of the dB difference in SRTs between the Base condition and the T, S, and T + S conditions. Figure 1(B) shows masking release for bilateral and bimodal CI users and for the three segregation cue conditions; mean masking release is shown in Table 3. In general, masking release was larger for bimodal than for bilateral CI users. For both bilateral and bimodal CI users, the largest masking release was observed for the T + S cue condition. For bilateral CI users, the smallest masking release was observed for the S condition; for bimodal CI users, mean masking release with the S condition was much higher, and comparable to that with the T + S condition. For bimodal CI users, mean masking release was smallest for the T condition. LMM analysis was performed on the masking release data, with group (bilateral, bimodal) and segregation cue (T, S, T + S) as fixed factors and participant as the random factor; complete results are shown in Table 3. Results showed significant effects of group [F(1, 17) = 4.93, p = 0.040] and segregation cue [F(2, 34) = 5.31, p = 0.010]; there was a significant interaction [F(2, 34) = 3.63, p = 0.037]. *Post hoc* Bonferroni-adjusted pairwise comparisons showed that masking release was significantly larger for bimodal than for bilateral CI users only for the S condition (p < 0.05), and significantly larger for the S and T + S conditions than for the T condition only for bimodal CI users (p < 0.05).

**Table 3.** Top: Masking release (mean ± standard deviation) relative to Baseline for the segregation cue conditions and CI groups. Bottom: Results from linear mixed model analysis of masking release data. Significant differences for pairwise comparisons are shown at right (adjustment for multiple comparisons: Bonferroni). BI = bilateral CI users; BM: bimodal CI users; T = Talker sex; S = Spatial; T + S: Talker sex + Spatial.

	Mean masking release (dB)		
	Segregation cue condition		
Group	T	S	T + S
BI	0.78 ± 2.03	0.19 ± 2.34	1.68 ± 2.02
BM	1.21 ± 1.65	3.62 ± 2.97	3.98 ± 3.47

Linear mixed model				
	dF, res	*F*	*p*	Post-hoc (p < 0.05)
Group	1, 17	4.93	0.040^a^	BM > BI
Cue	2, 34	5.31	0.010^a^	T + S > T, S
				S: BM > BI
Group × cue	2, 34	3.63	0.037^a^	BM: S, T + S > T

^a^ Significant effects.

## 4. Discussion

In contrast to our prediction and previous data observed in English-speaking CI users, Mandarin-speaking CI users significantly benefitted from spatial cues with bimodal listening but not with bilateral listening. Consistent with our prediction and previous data, the largest masking release was observed when both talker sex and spatial cues were available. However, the combined cue advantage differed between bilateral and bimodal CI users. For bilateral CI users, mean masking release with combined talker sex and spatial cues was 0.90 dB larger than that with talker sex cues alone, and 1.49 dB greater than that with spatial cues alone. For bimodal CI users, mean masking release with combined talker sex and spatial cues was 2.77 dB larger than that with talker sex cues alone, but only 0.36 dB larger than that with spatial cues alone. Thus, masking release with combined talker sex and spatial cues appeared to be largely driven by spatial cues in bimodal CI users.

Different from a related study with English-speaking CI users (Willis *et al.*, 2021), the present Mandarin-speaking CI users experienced substantial masking release with spatial cues, especially for bimodal CI users. Willis *et al.* (2021) reported negative masking release (i.e., interference) with spatial cues in English-speaking CI users (mean = −1.96 ± 0.64 dB across bilateral and bimodal CI users). Davis and Gifford (2018) also reported negative masking release in English-speaking bilateral CI users (mean = −0.42 dB) using a similar task but with 1-talker masker speech. The present Mandarin-speaking exhibited positive masking release with spatial cues (mean = 1.63 ± 3.08 dB across bilateral and bimodal CI users), especially for bimodal CI users (mean = 3.67 ± 2.97 dB).

**Fig. 2.**
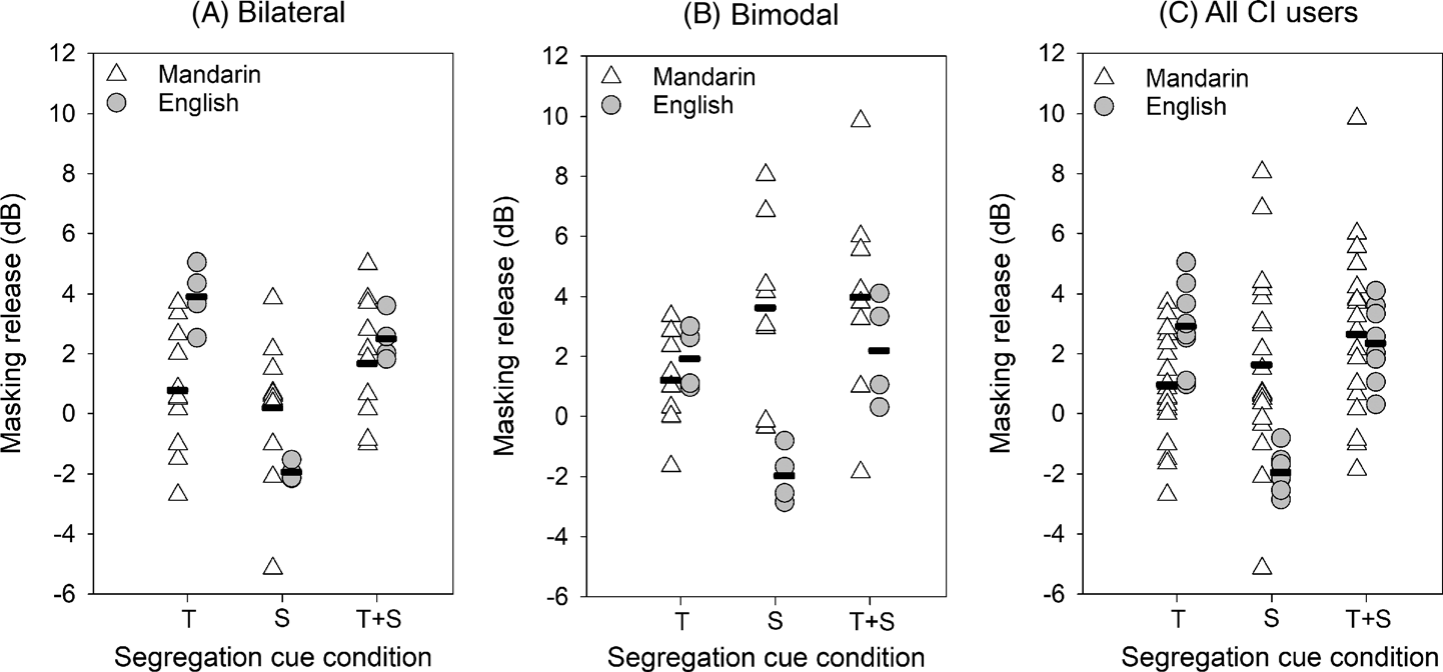
(A) Masking release for Talker sex (T), Spatial (S), and Talker sex + Spatial (T + S) cue conditions for Mandarin-speaking and English-speaking bilateral CI users. (B) Masking release for the T, S, and T + S cue conditions for Mandarin-speaking and English-speaking bimodal CI users. (C) Masking release for the T, S, and T + S cue conditions for Mandarin-speaking and English-speaking across bilateral and bimodal CI users. Masking release was calculated relative to the Base condition. In all panels, the horizontal dashes show mean performance. The Mandarin data are from the present study; the English data are from Willis *et al.* (2021).

Figure 2 shows masking release for the English- and Mandarin-speaking CI users for the three segregation cue conditions. Mean masking release with spatial cues was 2.13 dB greater for Mandarin-speaking than for English-speaking bilateral CI users [Fig. 2(A)], 5.59 dB greater for Mandarin-speaking than for English-speaking bimodal CI users [Fig. 2(B)], and 3.39 dB greater for Mandarin-speaking than for English-speaking participants, across CI users [Fig. 2(C)]. LMM analysis was performed on the masking release data from the present Mandarin-speaking CI users and the English-speaking CI users in Willis *et al.* (2021), with language (Mandarin, English), group (bilateral, bimodal), and segregation cue (T, S, T + S) as fixed factors and participant as the random factor; complete results are shown in Table 4. A significant effect was observed only for cue [F(2, 46) = 17.23, p < 0.001], but significant interactions were observed between language and cue [F(2, 46) = 19.52, p < 0.001], and between group and cue [F(2, 46) = 3.76, p = 0.031]. *Post hoc* Bonferroni-adjusted pairwise comparisons showed that masking release was significantly larger for Mandarin-speaking than for English speaking CI users with spatial cues (p < 0.05), but significantly larger for English speaking than for Mandarin-speaking CI users with talker sex cues. For Mandarin-speaking CI users, masking release was significantly larger with spatial cues alone or combined talker sex and spatial cues than with talker sex cues alone (p < 0.05). For English-speaking CI users, masking release progressively increased from spatial cues alone to combined talker sex and spatial cues to talker sex cues alone (p < 0.05). For bilateral CI users, masking release was greater with talker sex alone or combined talker sex and spatial cues than with spatial cues alone (p < 0.05). For bimodal CI users, masking release was greater with combined talker sex and spatial cues than with talker sex cues alone (p < 0.05).

**Table 4.** Results from linear mixed model analysis of masking release data from Mandarin-speaking (present study) and English-speaking CI users [from Willis *et al.* (2021)]. Significant differences for pairwise comparisons are shown at right (adjustment for multiple comparisons: Bonferroni). T = Talker sex; S = Spatial; T + S: Talker sex + Spatial; MAN = Mandarin-speaking CI users; ENG = English-speaking CI users; BI = bilateral CI users; BM: bimodal CI users.

Linear mixed model				
	dF, res	F	Sig.	Post-hoc (p < 0.05)
Language	1, 23	1.18	0.289	
Group	1, 23	0.75	0.396	
Cue	2, 46	17.23	<0.001^a^	T, TS > S
Language × group	1, 23	3.62	0.070	
Language × cue	2, 46	19.52	<0.001^a^	S: MAN > ENG; T: ENG > MAN
				MAN: TS, S > T
				ENG: T > TS > S
Group × cue	2, 46	3.76	0.031^a^	BI: T, TS > S
				BM: TS > T
Language × group × cue	2, 46	0.18	0.834	

^a^ Significant effects.

To further validate the benefit of combined use of a hearing aid and CI, three of the Mandarin-speaking bimodal CI users (BM6, BM7, BM8) were tested while listening with the CI alone. For these three participants, mean masking release with spatial cues was −1.03 ± 2.37 dB with CI-only listening, vs +0.85 ± 1.91 dB with bimodal listening. A paired t-test showed that masking release with spatial cues was significantly larger with bimodal than with CI-only listening [t(2) = −6.48, p = 0.023]. Willis *et al.* (2021) estimated the signal-to-noise ratio (SNR) for the co-located and spatially separated condition and found that the symmetrically placed masker sentences reduced the SNR by 1.2 dB, relative to the co-located maskers. This deficit was comparable to the negative masking release observed with the CI-only condition, suggesting that the increased (worsened) SRTs with spatial cues available were primarily driven by the reduced SNR in the spatially separated listening condition.

While intra- and inter-aural frequency mismatch has been proposed to explain the limited masking release with spatial cues (Willis *et al.*, 2021), it may not explain the difference in spatial masking release between Mandarin- and English-speaking CI users, especially for bimodal CI users. For English-speaking CI users, previous studies have shown no significant difference in spatial masking release between bimodal and bilateral CI users (Gifford *et al.*, 2014; Willis *et al.*, 2021). Gifford *et al.* (2018) found that under conditions of source location uncertainty, bilateral CI performance was better than bimodal performance when target speech was presented to the non-implanted ear, where performance was poorer than when speech was presented to the CI ear. Such an advantage may also be driven by the improved sound source localization with bilateral hearing over bimodal hearing (Dorman *et al.*, 2016). However, for the present Mandarin-speaking CI users, bimodal CI users exhibited significantly larger spatial masking release than did bilateral CI users. The better spatial masking release with bimodal than with bilateral CI users may be unique to tonal language speakers, as similar benefits of spatial cues have been observed in Mandarin-speaking CI users who combine acoustic and electric hearing. For single-sided deaf CI users, Chen *et al.* (2023) found an improvement of 4.8 dB in spatial masking release when the CI was added to the contralateral acoustic hearing.

The better spatial masking release with bimodal users may be partly due to the large F0 excursions associated with the lexical tones in Mandarin [e.g., Zhang *et al.* (2020)]. Previous studies have shown that dynamic changes in F0 can facilitate segregation of simultaneous streams of continuous speech [e.g., Summerfield and Culling (1992) and Divenyi *et al.* (1997)]. The F0 excursions in English are much smaller than in Mandarin (Zhang *et al.*, 2020). Without the large dynamic change in F0, spatial cues alone may not be sufficient for segregation due to the distorted binaural cues associated with the CI and hearing aids in English bimodal CI users. However, Mandarin-speaking bimodal CI users may be better able to combine the somewhat weak spatial cues and large dynamic changes in F0 for segregating the target form the competing speech, resulting in the better spatial masking release.

Interestingly, Mandarin-speaking CI users benefited less from talker sex cues than did English-speaking CI users in a related study (Willis *et al.*, 2021). Across bimodal and bilateral CI users, the mean masking release was 0.96 ± 1.84 dB for the present Mandarin-speaking CI users and 2.91 ± 1.44 dB for English-speaking CI users in Willis *et al.* (2021). The difference in masking release between Mandarin- and English-speaking CI users was significant for bilateral CI users [t(13) = −2.89, p = 0.003], but not for bimodal CI users [t(10) = −0.78, p = 0.384]. The talker sex masking release data were in contradiction to our prediction and to previous data reported in NH listeners. Due to the degraded spectral resolution with the CI, pitch perception and/or sensitivity to dynamic F0 cues is generally poorer in CI users than in NH listeners [e.g., Xie and Myers (2015) and Deroche *et al.* (2019)]. Still, the reduced benefit of talker sex cues in the present Mandarin-speaking bilateral CI users, relative to the English-speaking bilateral CI users in Willis *et al.* (2021) was quite surprising. It is possible that the reduced benefit may be due to a lack of improvement in tone recognition when talker sex cues were available, as the temporal envelope contour is the primary cue for tone recognition in Mandarin-speaking CI users (Fu and Zeng, 2000; Fu *et al.*, 1998). Also, the large dynamic changes in F0 associated with lexical tones may have obscured voice pitch differences between the male target and the female maskers (134–357 Hz). For Mandarin, F0s across all words ranged from 81 (10th percentile) to 199 Hz (90th percentile) for the male target, and from 134 (10th percentile) to 357 Hz (90th percentile) for the female target. For English, F0s across all words ranged from 90 (10th percentile) to 117 Hz (90th percentile) for the male target, and from 133 (10th percentile) to 189 Hz (90th percentile) for the female target. As such, there was much less overlap in F0 cues between target and masker speech for English than for Mandarin.

Different patterns were observed between Mandarin- and English-speaking CI users when both talker-sex sex and spatial cues were available. Though not significantly different, both English-speaking bilateral and bimodal CI users from Willis *et al.* (2021) exhibited lower mean masking release with combined talker sex and spatial cues than with talker sex cues alone, due to the poor (or even) negative masking release with spatial cues alone. This suggests that while spatial cues may have produced some interference, English-speaking CI users more strongly utilized talker sex cues when combined cues were available. For the present Mandarin-speaking bilateral CI users, there was no significant difference in masking release among the talker sex, spatial, and combined talker sex and spatial cue conditions (Table 3). For Mandarin-speaking bimodal CI users, masking release was significantly poorer with talker sex cues alone than with spatial cues alone or combined talker sex and spatial cues. Mean masking release was quite similar with talker sex cues alone or with combined talker sex and spatial cues, suggesting that poorer spatial masking release did not affect masking release with the combined cues.

While acoustic cues (e.g., large dynamic changes in F0) and better perception of F0 cues may be partly responsible for the better spatial masking release with Mandarin-speaking bimodal users, other factors may also contribute to the observed difference in spatial masking release between bilateral and bimodal CI user or between Mandarin and English. One factor is CI experience. In the present study, the mean CI experience was 5.5 years (range = 1.0–23.1 years) and 2.2 years (range = 1.3–4.0 years) for Mandarin-speaking bilateral and bimodal CI users, respectively. In Willis *et al.* (2021), the mean CI experience was 10.4 years (range = 2.5–26.1 years) and 2.7 years (range = 0.8–6.2 years) for English-speaking bilateral and bimodal CI users, respectively. A two-way analysis of variance (ANOVA) was performed on CI experience, with listening mode (bilateral, bimodal) and language (Mandarin, English) as factors. Results showed significant effects of listening mode [F(1,38) = 6.58, p = 0.014] but no significant effects of language [F(1,38) = 1.550, p = 0.221]; there was no significant interaction [F(1, 38) = 1.039, p = 0.315]. *Post hoc* Bonferroni-adjusted pairwise comparisons showed that English-speaking bilateral CI users had longer CI experience than did Mandarin-speaking bilateral CI users (p = 0.05). Bimodal CI users had significantly longer CI experience than did bilateral CI users for both Mandarin and English (p < 0.001), suggesting that the larger spatial masking release observed in Mandarin-speaking bimodal CI users is unlikely driven by CI experience.

Another large variable across different listening groups (bilateral vs bimodal) and language (Mandarin vs English) is the age at testing. In the present study, the mean age at testing was 23.6 years (range = 14–49 years) and 35.9 years (range =18–59 years) for bilateral and bimodal CI users, respectively. In Willis *et al.* (2021), the mean age at testing was 58.8 years (range = 36–70 years) and 64.0 years (range = 56–71 years) for bilateral and bimodal CI users, respectively. A two-way ANOVA was performed on age at testing, with listening mode (bilateral, bimodal) and language (Mandarin, English) as factors. Results showed significant effects of language [F(1,23) = 38.74, p < 0.001], but no significant effects of listening mode [F(1,23) = 2.985, p = 0.097]; there was no significant interaction [F(1, 23) = 0.484, p = 0.494]. *Post hoc* Bonferroni-adjusted pairwise comparisons showed that Mandarin-speaking CI users were significantly younger than English-speaking CI users (p < 0.001). A recent study showed that spatial release from informational masking declines with age (Zobel *et al.*, 2019). It is possible that worse spatial masking release observed in English-speaking CI users may be partly due to age. However, there was no significant difference in age at testing between bimodal and bilateral CI users, suggesting that age alone may not explain the difference in spatial masking release between Mandarin-speaking bimodal and bilateral CI users. A further study with age-matched comparison may be necessary to disentangle the role of age at testing and language on spatial masking release in bimodal CI users.

## Data Availability

The data that support the findings of this study are available from the corresponding author upon reasonable request.
